# Study protocol: Determinants of participation and quality of life of adolescents with cerebral palsy: a longitudinal study (SPARCLE2)

**DOI:** 10.1186/1471-2458-10-280

**Published:** 2010-05-26

**Authors:** Allan F Colver, Heather O Dickinson, SPARCLE group*

**Affiliations:** 1 Institute of Health and Society, Newcastle University, Newcastle upon Tyne, UK; 2 James Spence Building, Royal Victoria Infirmary, Queen Victoria Road, Newcastle upon Tyne NE1 4LP, UK

## Abstract

**Background:**

Children and adults with impairments such as cerebral palsy have lower participation in life situations than able-bodied people. Less is known about their subjective perception of their lives, called their quality of life.

During adolescence, rapid physical and psychological changes occur; although these may be more difficult for disabled than for able-bodied adolescents, little research has examined the lives of disabled adolescents.

In 2003-4 a European Union funded project, SPARCLE, visited 818 children aged 8-12 years with cerebral palsy, sampled from population-based registers in nine European regions. The quality of life reported by these disabled children was similar to that of the general population but their participation was lower; levels of participation varied between countries even for children with similar severity of cerebral palsy.

We are currently following up these children, now aged 13-17 years, to identify (i) to what extent contemporaneous factors (pain, impairment, psychological health and parental stress) predict their participation and quality of life, (ii) what factors modify how participation and quality of life at age 8-12 years are associated with participation and quality of life in adolescence, and (iii) whether differences between European countries in participation and quality of life can be explained by variations in environmental factors.

**Methods/Design:**

Trained researchers will visit families to administer questionnaires to capture the adolescents' type and severity of impairment, socio-demographic characteristics, participation, quality of life, psychological health, pain, environmental access and parental stress. We will use multivariable models (linear, logistic or ordinal) to assess how adolescent participation, quality of life, psychological health, pain, environmental access and parental stress, vary with impairment and socio-demographic characteristics and, where possible, how these outcomes compare with general population data. For participation and quality of life, longitudinal analyses will assess to what extent these are predicted by corresponding levels in childhood and what factors modify this relationship. Structural equation modelling will be used to identify indirect relationships mediated by other factors.

## Background

Children with impairments such as cerebral palsy are significantly disadvantaged;[1] in adulthood they continue to be disadvantaged with respect to employment and social relationships [2,3].

Much is now understood about factors which contribute to the disadvantage of **young **disabled children: many of the factors, such as services, support and physical aspects of the environment, are mediated through the family [4]. Far less is known about the teenage years; in particular there has been little attempt to unravel how the physical and psychological changes of adolescence are influenced by the person's impairments. Also there is little understanding of how participation and quality of life evolve as a child grows older, or how factors in childhood enable some disabled teenagers to adapt better than others.

### Conceptual framework

The Social Model of Disability [5] regards disability as resulting from the interaction between the individual and their environment rather than as intrinsic to the individual. The International Classification of Functioning, Disability and Health (ICF) [6], and its modification for children and young people ICF-CY [7] provide a powerful framework for operationalising this model. They define participation as involvement in life situations and examples of its nine domains are communication, domestic life and community life. Participation may be affected by the individual's impairments, their consequent activity limitations and contextual factors, both environmental and personal.

Formerly, concepts of quality of life (QoL) overlapped with those of handicap, function and activities of daily living [8]. QoL is now generally regarded as a subjective, self-reported concept [9]. WHO defines QoL as "an individual's perception of their position in life in the context of the cultural and value systems in which they live, and in relation to their goals, expectations, standards and concerns" [10].

The concepts of participation and QoL apply to everyone and it is especially important that they are considered for disabled people so that their needs, assessments and assistance are considered in the same framework as all people. Our own studies, Appendix paragraphs 1 and 5 and [11], show that lower QoL is only weakly related to severity of impairment whereas restricted participation is strongly related. Participation and QoL must therefore be assessed as separate but equally important outcomes - the former being objective, the latter subjective.

### Disabled adolescents

Adolescence is a developmental phase between childhood and adulthood that may last beyond age twenty [12]. A number of transitions occur such as moving from shared decision-making with parents to self-reliance; or from economic dependence to paid employment [13]. The adjustment is always challenging and often difficult, especially for disabled adolescents. Parents and care-givers may also find this time difficult as their role in the young person's life and their relationship with them changes. Growth and puberty will also interact with impairments; for example incontinence, previously tolerated, may adversely affect body image and emerging sexuality.

Health services for adolescents are recognised as inadequate [14-16]. As disabled adolescents move to care by adult teams, the holistic approach of child health, education and social services may be lost and available resources may be limited [17]. This may exacerbate a young person's psychological vulnerability.

### Participation and QoL of adolescents with cerebral palsy

Adolescents with cerebral palsy (CP) have a range of type and severity of impairments; in addition to problems with movement or posture, they usually have one or more additional impairments of vision, hearing, intellect, communication or behaviour. Some impairments may lead to secondary problems such as contractures, joint dislocation or chest infections. By studying adolescents with such a range of impairments, results can be generalised to disabled adolescents with other underlying conditions.

Adolescents with CP may be limited in their ability to participate fully in many school activities and social life compared with able-bodied peers [14,18]. The adolescent's participation may be affected by their type of CP and associated impairments [19,20]. Our studies show 8-12-year-old children with more severe impairments have lower participation (Appendix paragraph 5). Normal peer interactions, such as gossiping, fooling around and finding one's own solutions to problems may be prevented by the high level of adult surveillance experienced by some disabled children [21]. Pain is common in CP and is associated with lower participation Appendix paragraph 5 and [22] but the origins of the pain have not been studied systematically. Environmental barriers to participation include psychosocial pressures from family, school and community, financial difficulties, inadequate public services and physical barriers [23].

Studies of QoL of adolescents with CP give conflicting results, largely due to inadequate study design or because measures of function, mistakenly called and believed to be QoL, were used [24,25]. Our study of QoL of 8-12 year old children with CP showed that, on average, the QoL of those able to self-report was similar to that of the general population [11]. Although some impairments were associated with poorer QoL in some areas of their lives, their QoL was determined largely by other factors. It is not known whether QoL of children with CP changes as they reach adolescence but QoL of adolescents in the general population is reportedly lower than that of children, this effect being more marked in girls [26].

### The way forward

Our study aims to identify determinants of participation and QoL amenable to intervention and should therefore provide evidence to inform trials of interventions. Pain can be alleviated, psychological interventions can improve child mental health and parental stress; and the physical and social environment can be modified. We expect adolescent participation and QoL to be strongly predicted by corresponding scores in childhood; however, we will seek to identify the factors which modify this relationship, e.g. those factors associated with unfavourable outcomes in adolescents who had favourable outcomes in childhood.

Our specific objectives are to investigate in adolescents with cerebral palsy across Europe:

• How their participation and QoL compare with that of the general population and what factors predict differences.

• To what extent pain, impairment, psychological health and parental stress (all amenable to intervention) predict participation and QoL.

• What factors modify how participation and QoL at age 8-12 years are associated with participation and QoL in adolescence.

• Whether there are differences between European countries in participation and QoL; if so, whether these can be explained by variations in environmental factors.

### Basis for this collaboration and background work already conducted

This collaborative study builds on a programme of research undertaken by the SPARCLE group in nine European regions since 2000:

• Eight of these regions run a population-based register of children with cerebral palsy covering a defined geographical area. These regions belong to a collaboration funded by the European Union's Biomed 2 BMH4-983701 and DG SANCO QLG5-CT-2001-30133 and have reported on the epidemiology of CP across Europe [27]. The UK registers [28] have reported on life expectancy of people with CP in a study funded by the Medical Research Council [29].

• The SPARCLE group undertook a research project [30] funded by EU Research Framework 5 RF5 QLG5-CT-2002-00636 called "The influence of environmental factors on the participation and QoL of 8-12-year-old children with cerebral palsy in 9 European regions" and led by AC [11,23,30-47]. The main findings are summarised in Appendix 1 and full details of results and publications are located at http://www.ncl.ac/sparcle.

## Methods/Design

The primary design is a follow-up survey of 13-17-year-olds with CP from nine European regions. The children were randomly sampled from population-based registers, using an agreed classification of CP [27], at 8-12 years old when baseline data were gathered.

### Sample

The SPARCLE1 sample was established when the children were visited at 8-12 years [30,31]. In summary, 818 children born between 31st July 1991 and 1st April 1997 were visited, after sampling from eight population-based registers of children with cerebral palsy in six European countries. One further region recruited 75 children from multiple sources. The response rate of 63% was similar to that in surveys of similar design which target specific families and conduct face-to-face interviews [48]. In larger regions, sampling was stratified to ensure adequate numbers of children of all levels of impairment.

In order to minimise attrition, maximum effort will be made to re-interview every young person recruited for SPARCLE1, even if they have moved out of area. Nevertheless, on the basis of longitudinal cohort studies of children [49], we anticipate attrition of around 20%, yielding an achieved sample of about 650 participants across the nine regions, of whom 400 could self-report.

In order to maintain statistical power for cross-sectional analyses and possible further follow-up in adulthood, we will additionally sample from adolescents eligible for SPARCLE1, but not sampled then, in order to achieve a sample size similar to SPARCLE1. Assuming a non-response rate as in SPARCLE1, 270 further adolescents will need to be approached.

### Power calculation

The SPARCLE1 sample of 818 children had substantial statistical power: it allowed detailed multivariable models relating QoL and participation to psychological health, parental stress, impairment and socio-demographic factors [11,32,42,50,51].

Power calculations indicate that, in order to detect a difference of half a standard deviation (regarded as clinically important) between two sub-groups, at 90% power and 1% significance, we would require 120 adolescents in each group. A smaller sample of 90 adolescents in each group would be adequate if baseline (childhood) values were used as covariates, assuming a correlation of 0.5 between childhood and adolescent scores. Hence we anticipate that the achieved sample should be adequate to detect differences between major sub-groups of children, in both cross-sectional and longitudinal analyses; and even if comparisons are restricted to self-reporting children. Furthermore, even if there was 30% attrition at random, confidence intervals for multivariable models of factors associated with QoL of self-reporting children [11] would increase by about 20%, but all factors previously significant at the p < 0.01 level would remain significant at p < 0.05. For effective structural equation modelling with two to four factors, the recommended sample size is at least 200 [52], a sample size which again would be achieved among self-reporting children.

### Training of RAs

An RA will be appointed in each region to revisit the SPARCLE1 children, now adolescents aged 13-17 years. The RAs will attend a training workshop (see Table 1) to ensure quality, consistency between centres and motivation. Instruction will include the rationale for the study, study design, how to engage adolescents and administer the questionnaires, disability issues, good clinical practice and ethical requirements of the Declaration of Helsinki [53]. Following this, each RA will undertake five pilot visits and a second training workshop will address any difficulties encountered.

**Table 1:** Project timetable: milestones and workshops

MILESTONES	Description	Month
**1**	Ethics permissions in place	2
**2**	Completion of training of RAs and their pilot visits	4
**3**	Completion of agreed protocols for data analysis	14
**4**	Completion of visits to adolescents	14
**5**	Completion of data cleaning and scoring of questionnaires	16
**6**	Completion of development of analysis programs and testing on sample of data	18
**7**	Completion of initial analyses and drafts of papers	28
**8**	Completion of report	30
**WORKSHOPS**	**Description**	

**1**	Management group meeting	-3
**2**	Train RAs	2
**3**	Further meeting of RAs after pilot visit	4
**4**	Share expertise in statistical methodology and programs.	18
**5**	Finalise and agree results; scrutinise preliminary papers	28

### Visits

The RAs will visit at venues to suit the adolescents and parents. The questionnaires are self-completed but the RA will explain them, answer queries that arise and, for adolescents with motor or communication difficulties, assist or bring a non-parental interpreter of sign language or unclear speech.

### Instruments

As QoL concerns how a person perceives their life, it should be captured wherever possible by a self-report instrument, designed for and derived from work with the relevant age group. KIDSCREEN [54] is such an instrument developed from work with children and adolescents across Europe and used by the applicants in their earlier study [11,32,42]. Adolescents with severe learning difficulties (about one quarter of people with cerebral palsy) should be included in studies and their QoL inferred from reports by parents or other proxies.

For personal factors, we will capture: the adolescent's age, gender, psychological health and their self-efficacy or motivation, which is a distinct, concrete aspect of personality that has been well studied [55,56] and postulated to be a determinant of participation and QoL.

For contextual factors we will capture: the school and community and family environment family environment, including parental stress. In families of disabled children, parental stress is higher, especially if the child is severely impaired; this stress is associated with lower QoL in their children [32,57].

Table 2 lists the proposed instruments, the concepts captured, the relevant age range and whether general population data are available for comparison. It also shows the links with SPARCLE1 instruments. Because we are interested primarily in predictors of adolescent outcomes (rather than magnitude of change in outcomes) and parent-reported child factors may be valid predictors of adolescent outcomes, it is not essential that instruments are the same as in SPARCLE1 or reported by the same person. Where adolescents can self-report, they will complete instruments; where they cannot, parents will.

**Table 2:** Instruments to be used in SPARCLE2

SPARCLE1	SPARCLE2	Captures	Domains	Comments
Life-H [64,65]	Same instrument	Participation	Communication, Personal care, Home life, mealtimes, Mobility, Fitness, Recreation, Responsibility, Education, Community Life, Interpersonal relations	5-20 years
Frequency of participation questionnaire [51]	Modified instrument based on qualitative interviews with adolescent	Frequency of discretionary participation	31 items: Home life, Getting on with other people, Education, Work and finances, Community and political life, Recreation and leisure, Preparing for the future,	13-20 years General population data available
KIDSCREEN [54]	Same instrument	Quality of life	Psychological, Emotion, Social support, Home life, Self perception, Autonomy, School, Social acceptance, Finance, Physical well-being	8-18 years Data available from general population with socio-economic details
Strength and Difficulties Questionnaire (SDQ) [58]	Same instrument	Emotional health and behaviour	Emotional symptoms Conduct problems Hyperactivity Peer problems Pro-social behaviour	8-16 years. But can be used up to 19 years (Goodman Personal Communication) General population data available from some countries but socio-economic details not available
Parenting Stress Index Short Form (PSI-SF) [66]	Same instrument	Parental stress	Parental distress Parent-child dysfunctional interaction Difficult child PSI Life stress scale	Data available from general population from all SPARCLE countries but socio-economic details not available
Impairment	Same descriptors	Impairments	Type of cerebral palsy, Gross motor function, Upper limb function, Intelligence, Hearing, Vision, Communication, Feeding Seizures	This is descriptive
Background young person child and family factors	Same descriptors		Age, Sex, School, Siblings, Partner status, Parental educational qualifications, Parental employment status, Parental occupation	This is descriptive
Child Health Questionnaire [67]. Two questions on pain	Same questions	Pain severity and frequency		8-18 years Additional questions asked about location and cause of pain
	Pediatric Pain Questionnaire [68]	Pain location		13-18 years
	Dimensions of mastery questionnaire [56]	Motivation and self-efficacy	Total mastery score	14-18 years Data available from general population but socio-economic details not available
Environmental questionnaire [35,44].	Same questions	Availability of environmental factors required to facilitate participation	Physical home, Physical school, Physical community, Transport, Social support home, Social support community, Attitudinal schoolteachers and therapists, Attitudinal classmates	6-18 years

Where adolescent and parent may have important different perspectives such as for psychological health [58], each will be asked to complete the instrument. The parent **and **adolescent view of QoL of those who can self-report will both be captured in order to facilitate inferences about the QoL of adolescents who cannot self-report.

### Data management

Photocopies of completed questionnaires will be sent to Newcastle for double entry on a dedicated database. Data entry and validation will be continuous so that problems will be identified and addressed immediately rather than becoming apparent on completion of data collection. Programmes - already written for questionnaires used in SPARCLE1 - will be run to check missing data, ranges of responses and consistency between regions.

### Data analysis

In order to prevent bias due to "post hoc" analyses, protocols for statistical analysis will be pre-specified and agreed by month 14, milestone 3. Drop-out will be examined for relationships to observed factors such as age, gender, level of impairment and baseline levels of QoL, participation and parental stress [31]. As in SPARCLE1, the psychometric performance of the instruments (internal consistency, convergent/divergent validity, confirmatory factor analyses) will be compared with properties reported by their developers. For the adolescent's participation, QoL, psychological health and parental stress, domain scores will be compared with general population data, adjusting where possible for socio-demographic characteristics [11].

**Figure 1:**
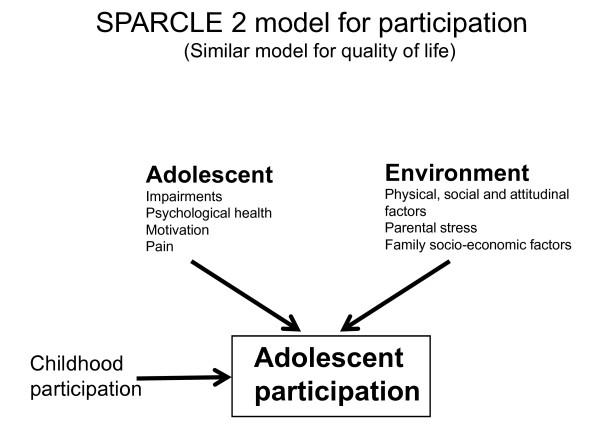
**Model of predictors of adolescent participation**.

Figure 1 shows diagrammatically the essence of the model we shall use.

Whilst cross-sectional analyses will compare disabled adolescents with the general population of the same age, most analyses will utilise the strength of longitudinal design to understand not only the contemporaneous but also the childhood predictors of adolescent participation and QoL.

• **A **For the adolescent's participation, QoL, psychological health, pain, parental stress and environmental access, multivariable models (linear, logistic or ordinal) will be used to assess how scores on each domain vary with type and level of impairment, and adolescent and family socio-demographic characteristics [11,32,50,51].

• **B **For participation and QoL, longitudinal analyses will assess to what extent these are predicted by corresponding scores in childhood and what factors modify this relationship. Sensitivity analyses will assess the possible effect of differential attrition [59]. These analyses will model the non-response mechanism on the basis of plausible values informed by clinical expertise, with weights corresponding to the inverse probability of non-response [60].

The models of participation and QoL generated by points A and B above will then be combined in unified models for each outcome using structural equation modelling (SEM). These will model the latent traits captured by the various instruments, (including the participation and QoL in childhood), to predict adolescent participation and QoL [61,62]. SEM can quantify both direct relationships between latent traits, the effects of factors that modify these relationships, and also indirect relationships mediated by other factors. Plausible models will be informed by clinical expertise. The longitudinal nature of the study, with data at two time points on each child, may facilitate identification of causal pathways [63].

Primary analyses will use Stata, based on programs written for SPARCLE1; SEM will use LISREL or Mplus.

### Project management

The responsibility for delivering on all objectives lies with the project management group which will comprise the nine scientific leads, the senior research associate in Newcastle and statisticians in Newcastle and Toulouse. The group will meet during the project's workshops (see Table 1). AFC will co-ordinate the project.

The central co-ordination, data collation and much statistical analysis will take place in Newcastle, as in SPARCLE1.

An external advisory group will meet at the beginning of the project and soon after data analysis has begun to monitor progress. Membership will comprise a young adult with cerebral palsy, paediatrician with adolescent expertise, medical statistician, research paediatric orthotist and chief executive of a voluntary organisation.

### Ethics

For those in SPARCLE1, we already have permission to contact the family for a further study. For those not approached before, the method of SPARCLE1 will be used where a health professional who knows the child, such as General Practitioner or Paediatrician, will approach the family.

We will strive to approach the adolescents in such a way that they can make an informed decision about whether to join the study. For those under 16 years (18 years in France), parental consent must be obtained in addition to that of the adolescent. For those over 16, we will not seek parental consent but will take seriously a parent who did not want their child to join the study.

If the adolescent becomes distressed by questions on sensitive issues, the RA cannot provide support but will facilitate contact with general practitioner or psychological service.

If the RA is concerned that the adolescent might be being abused, action in a person over 18 must be with the adolescent's agreement. If under 18, the RA will first liaise with the scientific lead in each region to ensure statutory procedures are followed.

The safety of the RAs will be maximised by ensuring visits are to home or school; by providing a mobile phone and requiring the RA to tell the office their expected time of return.

The National Research Ethics Service, Newcastle and North Tyneside Research Ethics Committee, approved the project: 09/H0906/4.

### Dissemination

The findings of the study will be disseminated in a range of formats:

• Academic publications. Our output from SPARCLE1 [11,23,30-47]demonstrates the importance we attach to this and our ability to deliver.

• Feedback to young people and parents, as already undertaken in SPARCLE1.

• Meetings of European Academy of Childhood Disability, Royal College of Paediatrics and Child Health and meetings around QoL and adolescence.

• An official report with executive summary which will be presented to National Governments, the European Commission and the European Parliament, as in SPARCLE1.

## Discussion

This study will increase understanding of how to improve the life and health of disabled adolescents - a neglected group in which few epidemiological studies have been undertaken.

Health is a broad concept, defined by WHO [10] as a state of complete physical, mental and social wellbeing. This is especially relevant to disabled people who often have no disease and impairments that are relatively stable. Although much medical research aims to prevent disease and impairments and treat disease, for disabled people improved health may be only marginally influenced by medical intervention; participation in social and family life and effective transition to adulthood, which are important components of health, may respond more readily to social and environmental intervention.

The UN Conventions on Rights of the Child, and Rights of Persons with Disabilities recognise the rights of disabled children to participate fully in life and express their views. By capturing quality of life as a key outcome, our study will allow disabled adolescents a voice and findings should influence attitudes towards them.

In our earlier study of 8-12-year-old children with cerebral palsy, there were marked differences in child participation between countries, after controlling for severity of impairment. This suggests the environment is more facilitatory in some countries than others. If longitudinal analyses confirm these associations, they would provide strong evidence for advocacy and policy development in health, education and social sectors. Associations of participation and QoL with pain, psychological health and parental stress will also be studied; as all are amenable to intervention, results should inform trials of interventions.

## Competing interests

The authors declare that they have no competing interests.

## Authors' contributions

The protocol was developed and agreed jointly by the members of the SPARCLE group in a series of workshops. AFC and HOD drafted the manuscript. All members of the SPARCLE group contributed to the design of the study and this manuscript and all have approved the final manuscript.

## Appendix. Main findings of SPARCLE1 - Study of participation and quality of life of 8-12 year old Children with Cerebral Palsy Living in Europe

The main findings to date are:

1. Children with CP who were able to report their own QoL had, on average, similar QoL to children of the same age in the general population. Although some impairments were associated with lower QoL in some areas of life of the children with CP, these factors accounted for less than 10% of the variation in QoL of disabled children and therefore their QoL must be largely determined by other factors such as those that influence QoL in non-disabled children.

2. Pain is common and significantly associated with lower QoL.

3. When the child's report of their QoL is compared to what their parents think it is, parents usually underestimate their child's QoL.

4. For children with intellectual impairment, parental reports indicate that pain is associated with poorer QoL on some domains; restricted mobility with reduced physical well being and autonomy. However restricted mobility and intellectual impairment were associated with better QoL on other domains. Parents who are more stressed tend to report poorer QoL in their child across all domains.

5. The participation of children with cerebral palsy is considerably lower than that of children in the general population. This is more pronounced for those with severe motor impairment; and for those with more impairments. Pain is also associated with reduced participation.

6. For children with similar levels of impairment, participation varies significantly between countries.

7. Children with cerebral palsy have more psychological problems than the general population. Among children with learning difficulties, these psychological problems are commoner in children with the milder physical impairments.

8. Parents of children with cerebral palsy experience more stress than parents in general. Stress tends to be greater for parents whose children have more severe cerebral palsy. Parental stress is associated with lower child-reported QoL on five of the ten QoL domains.

9. The seven EU countries in the study vary considerably in their attitudinal environment and in environmental law, regulation and social and physical provision for disabled children. These differences are summarised in a published report commissioned by the SPARCLE project.

10. An environmental questionnaire was developed. The degree to which families and their disabled children receive the environmental support they need varies greatly. Children with less need are more likely to have their needs met; and certain factors such as wheelchairs are provided much more often than for example communication aids. There is also striking variation across the European EU regions in the extent to which needed environmental features are available.

11. We think, though these analyses are not completed, that some aspects of the environment concerning adaptations, access to buildings, transport, schooling etc are are strongly associated with better participation; but effects on QoL are much weaker.

12. At the end of the home visit, we asked children to indicate on paper, confidentially and anonymously, what they felt about being asked all the questions; and what they thought about the research associate who visited them. Two centres did not administer it. Of 340 children who could complete the "smiley face" options, only 12 children were unhappy about being asked the questions and only four children were unhappy about the person who visited them.

## Pre-publication history

The pre-publication history for this paper can be accessed here:

http://www.biomedcentral.com/1471-2458/10/280/prepub

## References

[B1] Sloper P Turner S Service needs of families of children with severe physical disability Child Care Health Dev 1992 18 259 282 10.1111/j.1365-2214.1992.tb00359.x 1394855

[B2] Michelsen SI Uldall P Kejs AMT Madsen M Education and employment prospects in cerebral palsy Dev Med Child Neurol 2005 47 511 517 10.1017/S0012162205001015 16108450

[B3] Michelsen SI Uldall P Hansen T Madsen M Social integration of adults with cerebral palsy Dev Med Child Neurol 2006 48 643 649 10.1017/S0012162206001368 16836775

[B4] Law M Hanna S King G Hurley P King S Kertoy M Rosenbaum P Factors affecting family-centred service delivery for children with disabilities Child Care Health Dev 2003 29 357 366 10.1046/j.1365-2214.2003.00351.x 12904243

[B5] Oliver M The Politics of Disablement 1990 London: Macmillan

[B6] World Health Organisation International classification of functioning, disability and health Book International classification of functioning, disability and health 2001 WHO, Geneva

[B7] World Health Organisation World Health Organisation Classification of Functioning, Disability and Health. Children and Youth Version Book World Health Organisation Classification of Functioning, Disability and Health. Children and Youth Version 2007 WHO, Geneva

[B8] Muldoon MF Barger SD Flory JD Manuck SB What are quality of life measurements measuring? BMJ 1998 316 542 545 9501721 10.1136/bmj.316.7130.542 PMC2665651

[B9] Zekovic B Renwick R Quality of life for children and adolescents with developmental disabilities: review of conceptual and methodological issues relevant to public policy Disabil Soc 2003 18 19 34 10.1080/713662199

[B10] WHOQOL The World Health Organization quality of life assessment: position paper from the World Health Organization Soc Sci Med 1995 41 1403 1409 10.1016/0277-9536(95)00112-K 8560308

[B11] Dickinson HO Parkinson KN Ravens-Sieberer U Schirripa G Thyen U Arnaud C Beckung E Fauconnier J McManus V Michelsen SI Self-reported quality of life of 8-12-year-old children with cerebral palsy: a cross-sectional European study Lancet 2007 369 2171 2178 10.1016/S0140-6736(07)61013-7 17604799

[B12] Fulginiti VA What is pediatrics?: prenatal medicine to young adult care Am J Dis Child 1992 146 17 18 1445504

[B13] Schidlow DV Fiel SB Life beyond pediatrics. Transition of chronically ill adolescents from pediatric to adult health care systems Med Clin North Am 1990 74 1113 1120 2201847 10.1016/s0025-7125(16)30505-3

[B14] Stevenson CJ Pharoah PO Stevenson R Cerebral palsy--the transition from youth to adulthood Dev Med Child Neurol 1997 39 336 342 9236701 10.1111/j.1469-8749.1997.tb07441.x

[B15] Bowes G Sinnema G Suris JC Buhlmann U Transition health services for youth with disabilities: a global perspective J Adolesc Health 1995 17 23 31 10.1016/1054-139X(95)00076-5 7578158

[B16] Intercollegiate Working Party on Adolescent Health Bridging the gaps: health care for adolescents Book Bridging the gaps: health care for adolescents 2003 London: Royal College of Paediatrics and Child Health

[B17] Ko B McEnery G The needs of physically disabled young people during transition to adult services Child Care Health Dev 2004 30 317 323 10.1111/j.1365-2214.2004.00422.x 15191421

[B18] Blum RW Overview of transition issues for youth with disabilities Pediatrician 1991 18 101 104 1832222

[B19] Hammal D Jarvis S Colver A Participation of children with cerebral palsy is influenced by where they live Dev Med Child Neurol 2004 46 292 298 10.1017/S0012162204000489 15132258

[B20] Beckung E Hagberg G Neuroimpairments, activity limitations, and participation restrictions in children with cerebral palsy Dev Med Child Neurol 2002 44 309 316 10.1017/S0012162201002134 12033716

[B21] Watson N Shakespeare T Cunningham-Burley S Barnes C Life as a disabled child: A qualitative study of young people's experience and perspectives 1999 Department of Nursing Studies, University of Edinburgh: Economic and Social Science Research Council

[B22] Houlihan CM O'Donnell M Conaway M Stevenson RD Bodily pain and health-related quality of life in children with cerebral palsy Dev Med Child Neurol 2004 46 305 310 10.1017/S0012162204000507 15132260

[B23] Mihaylov SI Jarvis S Colver A Beresford B Identification and description of environmental factors that influence participation of children with cerebral palsy Dev Med Child Neurol 2004 46 299 304 10.1017/S0012162204000490 15132259

[B24] Rosenbaum PL Livingston MH Palisano RJ Galuppi BE Russell DJ Quality of life and health-related quality of life of adolescents with cerebral palsy Dev Med Child Neurol 2007 49 516 521 10.1111/j.1469-8749.2007.00516.x 17593124

[B25] Livingston MH Rosenbaum PL Russell DJ Palisano RJ Quality of life among adolescents with cerebral palsy: what does the literature tell us? Dev Med Child Neurol 2007 49 225 231 10.1111/j.1469-8749.2007.00225.x 17355481

[B26] Bisegger C Cloetta B von Rueden U Abel T Ravens-Sieberer U Health-related quality of life: gender differences in childhood and adolescence Soz Praventivmed 2005 50 281 291 10.1007/s00038-005-4094-2 16300172

[B27] SCPE Prevalence and characteristics of children with cerebral palsy in Europe Dev Med Child Neurol 2002 44 633 640 12227618

[B28] Surman G Bonellie S Chalmers J Colver A Dolk H Hemming K King A Kurinczuk JJ Parkes J Platt MJ UKCP: a collaborative network of cerebral palsy registers in the United Kingdom J Public Health (Oxf) 2006 28 148 156 10.1093/pubmed/fdi087 16556625

[B29] Hemming K Hutton JL Colver A Platt MJ Regional variation in survival of people with cerebral palsy in the United Kingdom Pediatrics 2005 116 1383 1390 10.1542/peds.2005-0259 16322162

[B30] Colver A Study protocol: SPARCLE - a multi-centre European study of the relationship of environment to participation and quality of life of children with cerebral palsy BMC Public Health 2006 6 105 10.1186/1471-2458-6-105 16638126 PMC1459857

[B31] Dickinson H Parkinson K McManus V Arnaud C Beckung E Fauconnier J Michelsen SI Parkes J Schirripa G Thyen U Colver A Assessment of data quality in a multi-centre cross-sectional study of participation and quality of life of children with cerebral palsy BMC Public Health 2006 6 273 10.1186/1471-2458-6-273 17087828 PMC1636041

[B32] Arnaud C White-Koning M Michelsen SI Parkes J Parkinson K Thyen U Beckung E Dickinson HO Fauconnier J Marcelli M Parent-reported quality of life of children with cerebral palsy in Europe Pediatrics 2008 121 54 64 10.1542/peds.2007-0854 18166557

[B33] Parkes J White-Koning M Dickinson HO Thyen U Arnaud C Beckung E Fauconnier J Marcelli M McManus V Michelsen SI Psychological problems in children with cerebral palsy: a cross-sectional European study J Child Psychol Psychiatry 2008 49 405 413 10.1111/j.1469-7610.2007.01845.x 18081767

[B34] Michelsen SI Flachs E Uldall P Eriksen E McManus V Parkes J Parkinson KN Thyen U Arnaud C Beckung E Frequency of participation of 8-12-year-old children with cerebral palsy; a multi-centre cross-sectional European study Eur J Paediatr Neurol 2009 13 165 177 10.1016/j.ejpn.2008.03.005 18571944

[B35] Dickinson HO Colver A Quantifying the physical, social and attitudinal environment of children with cerebral palsy Disabil Rehabil 2010 in press 10.3109/09638288.2010.485668 20455710

[B36] Lawlor K Mihaylov S Welsh B Jarvis S Colver A A qualitative study of the physical, social and attitudinal environments influencing the participation of children with cerebral palsy in northeast England Pediatr Rehabil 2006 9 219 228 17050400 10.1080/13638490500235649

[B37] McConachie H Colver AF Forsyth RJ Jarvis SN Parkinson KN Participation of disabled children: how should it be characterised and measured? Disabil Rehabil 2006 28 1157 1164 10.1080/09638280500534507 16966237

[B38] McManus V Michelsen S Parkinson K Colver A Beckung E Pez O Caravale B Discussion groups with parents of children with cerebral palsy in Europe designed to assist development of a relevant measure of environment Child Care Health Dev 2006 32 185 192 10.1111/j.1365-2214.2006.00601.x 16441853

[B39] Tisdall K National contextual factors affecting the lives of disabled children in Denmark, France, Germany, Ireland, Italy, Sweden and UK (England and Northern Ireland) Book National contextual factors affecting the lives of disabled children in Denmark, France, Germany, Ireland, Italy, Sweden and UK (England and Northern Ireland) 2006 1 Newcastle: Newcastle University http://www.ncl.ac.uk/sparcle/Publications_files/WebVol1.pdf

[B40] Tisdall K National contextual factors affecting the lives of disabled children in Denmark, France, Germany, Ireland, Italy, Sweden and UK (England and Northern Ireland) Book National contextual factors affecting the lives of disabled children in Denmark, France, Germany, Ireland, Italy, Sweden and UK (England and Northern Ireland) 2006 2 Newcastle: Newcastle University http://www.ncl.ac.uk/sparcle/Publications_files/WebVol2.pdf

[B41] White-Koning M Arnaud C Bourdet-Loubere S Bazex H Colver A Grandjean H Subjective quality of life in children with intellectual impairment - how can it be assessed? Dev Med Child Neurol 2005 47 281 285 10.1017/S0012162205000526 15832552

[B42] White-Koning M Arnaud C Dickinson HO Thyen U Beckung E Fauconnier J McManus V Michelsen SI Parkes J Parkinson K Determinants of child-parent agreement in quality-of-life reports: a European study of children with cerebral palsy Pediatrics 2007 120 e804 814 10.1542/peds.2006-3272 17908738

[B43] Young B Rice H Dixon-Woods M Colver AF Parkinson KN A qualitative study of the health-related quality of life of disabled children Dev Med Child Neurol 2007 49 660 665 10.1111/j.1469-8749.2007.00660.x 17718821

[B44] Colver AF Dickinson HO Access of children with cerebral palsy to the physical, social and attitudinal environment they need: a cross-sectional European study Disabil Rehabil 2010 in press 20446803 10.3109/09638288.2010.485669

[B45] Erhart M Ravens-Sieberer U Dickinson HO Colver A Rasch measurement properties of the KIDSCREEN quality of life instrument in children with cerebral palsy and differential item functioning between children with and without cerebral palsy Value Health 2009 in press 19490565 10.1111/j.1524-4733.2009.00508.x

[B46] Carlsson M Olsson I Hagberg G Beckung E Behaviour in children with cerebral palsy with and without epilepsy Dev Med Child Neurol 2008 50 784 789 10.1111/j.1469-8749.2008.03090.x 18834391

[B47] McCullough N Parkes J White-Koning M Beckung E Colver A Reliability and validity of the Child Health QuestionnairePF-50 for European children with cerebral palsy J Pediatr Psychol 2009 34 41 50 10.1093/jpepsy/jsn048 18499739

[B48] Green H McGinnity A Meltzer H Ford T Goodman R Mental Health of Children and Young People in Great Britain 2004 2005 London: Office for National Statistics

[B49] Centre for Longitudinal Studies 2008 http://www.cls.ioe.ac.uk/publications.asp?section=0001000100060001

[B50] Parkes J White-Koning M Dickinson HO Thyen U Arnaud C Beckung E Fauconnier J Marcelli M McManus V Michelsen SI Psychological problems in children with cerebral palsy: a cross-sectional European study J Child Psychol Psychiatry 2008 49 4 405 13 10.1111/j.1469-7610.2007.01845.x 18081767

[B51] Michelsen SI Flachs E Uldall P Eriksen E McManus V Parkes J Parkinson K Thyen U Arnaud C Beckung E Frequency of participation of 8-12-year-old children with cerebral palsy; a multi-centre cross-sectional European study Eur J Paediatr Neurol 2009 13 165 77 10.1016/j.ejpn.2008.03.005 18571944

[B52] Loehlin J Latent variable models 1992 Hillsdale, NJ, Erlbaum Publishers

[B53] Williams JR The promise and limits of international bioethics: lessons from the recent revision of the Declaration of Helsinki J Int Bioethique 2004 15 31 42 10.3917/jib.151.0031 15835066

[B54] Ravens-Sieberer U Gosch A Rajmil L Erhart M Bruil J Duer W Auquier P Power M Abel T Czemy L KIDSCREEN-52 quality-of-life measure for children and adolescents Expert Review of Pharmacoeconom Outcomes Res 2005 5 353 364 10.1586/14737167.5.3.353 19807604

[B55] Thelen E Smith L A dynamic systems approach to the development of cognition and action 1994 Cambridge, Massachusetts: MIT Press

[B56] Morgan G Leech N Barrett K Busch-Rossnagel N Harmon R The dimensions of mastery questionnaire (manual) 2000 Colorado State University

[B57] Manuel J Naughton MJ Balkrishnan R Paterson Smith B Koman LA Stress and adaptation in mothers of children with cerebral palsy J Pediatr Psychol 2003 28 197 201 10.1093/jpepsy/jsg007 12654945

[B58] Goodman R Psychometric properties of the Strengths and Difficulties Questionnaire J Am Acad Child Adolesc Psychiatry 2001 40 1337 1345 10.1097/00004583-200111000-00015 11699809

[B59] Molenberghs G What to do with missing data? J R Stat Soc A 2007 170 861 863 10.1111/j.1467-985X.2007.00504.x

[B60] Rotnitzky A Robins JM Scharfstein DO Semiparametric regression for repeated outcomes with nonignorable nonresponse J Am Stat Assoc 1998 93 1321 1339 10.2307/2670049

[B61] Kline RB Principles and practice of structural equation modeling 2005 Second New York, Guilford Press

[B62] Byrne BM Structural equation modeling with LISREL, PRELIS and SIMPLIS: basic concepts, applications and programming 1998 Mahwah, NJ, Erlbaum

[B63] Dunn G Everitt B Pickles A Modelling covariances and latent variables using EQS 1993 London: Chapman & Hall/CRC

[B64] Lepage C Noreau L Bernard PM Fougeyrollas P Profile of handicap situations in children with cerebral palsy Scand J Rehabil Med 1998 30 263 272 10.1080/003655098444011 9825391

[B65] Noreau L Lepage C Boissiere L Picard R Fougeyrollas P Mathieu J Desmarais G Nadeau L Measuring participation in children with disabilities using the Assessment of Life Habits Dev Med Child Neurol 2007 49 666 671 10.1111/j.1469-8749.2007.00666.x 17718822

[B66] Abidin R Parenting Stress Index Professional Manual 1995 3 USA: Psychological Assessment Resources Inc

[B67] Landgraf J Abetz L Ware JE Child Health Questionnaire (CHQ): a User's Manual, Second Printing 1999 Boston, MA: HealthAct

[B68] Varni JW Pediatric Pain Questionnaire - Teen Form Book Pediatric Pain Questionnaire - Teen Form 1998 City: LICENSOR Mapi Research Trust

